# Activity and Social Responsibility in the Discourse on Health Care, Long-Term Care and Welfare Services for Older Immigrants

**DOI:** 10.1155/2021/5241396

**Published:** 2021-07-30

**Authors:** Andrea Goettler

**Affiliations:** Chair of Sociology of Diversity, Department of Sport and Health Sciences, Technical University of Munich, 80992 Munich, Germany

## Abstract

Ageing well has been associated with the responsibility to age actively, successfully, or healthily in public and research discourses. This connection of individual responsibility with ageing has been criticised in Social Gerontology for neglecting the access to social, economic, and health resources. This paper investigates (individual) responsibility, informal support, and public initiatives in discourses on older immigrants in Germany. The research framework employs a sociology of knowledge approach to discourse, which guided the discourse analysis of German policy reports, guidelines and handbooks on ageing and migration from 2000 to 2019 (43 documents in total). The results reveal that besides public initiatives concerning long-term care, health promotion, and social services, informal solutions through social networks are frequently emphasised in the data. The focus, thereby, is on long-term care, which is presented as a responsibility of the extended family. Thus, resources are situated in the family, social networks, and ethnic group, which should be opened and connected with public services; however, the focus is shifting from older immigrants towards local municipalities. This study provides a discourse perspective on the construction of resources and challenges for older immigrants concerning health, care, and social services and offers an assessment of the cultural and integrating/excluding qualities in active ageing discourses.

## 1. Introduction

Demographic change has led to increasing longevity and changes in the age structures on a global scale. These trends also affect the age distribution of migrant populations and have led to an increase in older migrants in Europe [1, 2]. A common response to this demographic change has been the promotion of active ageing to ensure healthy and independent older lives [3]. However, to develop active ageing and its potential, the concept must include the diversity of the ageing population, which is particularly important given the criticism and developments of ageing policies and discourses. Active ageing has been widely discussed and criticised for the connection of a positive image of old age with activity and health, the individual responsibility to stay active and healthy, and the neglect of socioeconomic and environmental factors [4]. Following these criticisms, it is important to evaluate active ageing regarding minority and low-resource populations, where structural determinants define ageing well [5]. While research has addressed the meanings and potentials of active ageing for older migrants [6, 7], the connection between migration and ageing discourses and the resulting construction of active ageing has not been investigated yet. Older immigrants are a growing population group in Germany, who constitute 12% of the population above 65 in 2019 [8] and who experience more health risks and financial insecurities compared to the autochthonous population [9]. While the term immigrant emphasises the nation-state borders of migration, I will specifically refer to older immigrants in the context of Germany to highlight the permanence of immigrants, especially in contrast to the commonly used phrased “guest worker” in Germany [11, 12], and to highlight the immigration state's responsibilities in welfare services for older immigrants. I will use the term migrant when not specifically referring to older immigrants in Germany. This paper explores (individual) responsibility, informal support, and public initiatives in the context of ageing immigrants in Germany and how active ageing is conceptualised regarding migration background.

The discourse on active ageing impacts our understanding of ageing and older age [10]. Hence, the discourse on older age is marked by an emphasis on individual responsibility, an expectation to stay active and healthy and to provide a valuable contribution to society in old age. This understanding of older age also affects welfare services and the construction of support structures for the older population. Thus, this study analyses the discourse on older immigrants in Germany in health promotion, long-term care, social services, and the welfare sector. The discourse analysis is carried out using the sociology of knowledge approach to discourse (SKAD) to examine how active ageing is conceptualised regarding migrants. The discourse arena comprises the macro- and meso-level examining policy documents, reports of health or care institutions, migrant organisations and political foundations, and articles from health- or migrant-related magazines. The focus, thereby, is on social and health-related public services, especially health promotion initiatives, ambulatory and stationary long-term care, social service centres for older people, and welfare services. This research provides a novel analysis of the active ageing discourse at the intersection of ageing and migration. The results display that active ageing is a dynamic framework that is constructed differently in the context of migration in Germany.

## 2. Discourses on Ageing Well

Society's expectations of ageing and older age are under constant negotiation and have significantly changed over the past decades. Old age as frail, sick, and dependant has been at least partially replaced by the idea of active and socially engaged older age. Yet, Angus and Reeves [13] remark that despite the emphasis on the healthy, independent, and self-responsible older individual, the contrasting image of dependant older age persists at the same time. Furthermore, the promotion of activity, health, and social participation in older age is shaped by an emphasis on the challenges of the demographic change [10, 14]. With lengthening life expectancies, the sustainability of pension schemes came under threat, which has led to a redefinition of retirement age as a time of activity, productivity, and opportunity. The policy objective to retain individual independence through better health and social involvement addresses both individual well-being as well as collective welfare [15]. This led to the promotion of a positive image of ageing, which is described in various ways, ranging from successful, productive, active, or healthy ageing. While these concepts overlap to a large extent, they also highlight different components: successful ageing predominantly focuses on physical functioning, productive ageing on economic contributions, whereas active and healthy ageing is more broadly understood [5]. Healthy ageing is the current main approach of the WHO, emphasised in the current Decade of Healthy Ageing 2020-2030 and defined as “developing and maintaining the functional ability that enables well-being in older age” ([16], 3). Active ageing is the concept most widely used, ranging from uni-dimensional approaches to definitions, which include behavioural and structural aspects, similar to healthy ageing. It is also the most prominent wording in age-related policies and presents a more holistic approach, which considers the life course and structural determinants of health [17].

Active ageing has first been introduced in the 1990s through the promotion of health and activity [17]. Since then, European states have adopted active ageing policies as a response to preceding age-discriminating descriptions of frail and passive old age [17]. It is rooted in two different discourses that have become increasingly interlinked. Based on the broader definition of the WHO, active ageing (or healthy ageing since 2015) emphasises general well-being, with a focus on physical and mental health and social participation [18]. However, other definitions have centred on the productivity aspects of retirement [10, 17, 19]. The connection of these different policies has led to productivity and social participation becoming the main tools for achieving improved well-being [20]. Although active ageing aims to improve the image of older populations from frail and passive to active and healthy, this call for activity has received substantial critique [4, 5, 10, 21]. First, through an emphasis on activity and productivity, a positive image of older age is always connected with activity and health as indicators. This poses the questions of what counts as an activity, especially, as active ageing discourse mainly emphasises actions valuable for the general society [5, 22, 23]. Second, highlighting the value for society comes with the responsibility and imperative for the individual to do their best in older age. To contribute to society, in form of productivity or social participation, is conceptualised as a necessity for an ageing population [10]. Thirdly, active ageing has been criticised for taking for granted financial, social, and health resources. Older people in low-resource situations are thus at risk of further marginalisation by not complying with the normative standard of ageing actively [23, 24]. This focus on health, responsibility, and resources has led to a dismissal of vulnerable populations in the active ageing context.

As a response to the overshadowing discourse on what older age should look like, there exists a vast amount of research on older people's experience-based perspectives on what ageing well means to them. In general, older people value a diversity of components emphasising physical, mental, social function, independence, and well-being as well as (social) engagement, positive attitude, family, adaptation, financial security, personal growth, and spirituality [25–27]. All studies confirm that ageing well—to use a general term—is multidimensional, complex, and culturally specific.

### 2.1. Ageing Discourses in the Context of Older Migrants

The determinants of health influencing ageing processes resemble across populations, yet migration has been linked to health risks in older age [28]. Despite generally good health during or after migrations, the health of migrants seems to decrease with time spent in the destination country [29]. Often health risks are caused by disadvantageous socioeconomic positions, language barriers, low health literacy, psychological vulnerability, and discrimination. Therefore, older migrants on average experience worse health outcomes and an earlier onset of age-related diseases [28]. Health risks are increasingly recognised in the policy context of active ageing, which aims to address the heterogenous life course aspects of older migrants [30, 31]. Bolzman and Kaeser [6] investigate the gap between the normative concept of active ageing both in its productive and holistic understanding and the reality of older migrants among the older Spanish and Italian population in Switzerland. They summarise that it is important to provide the societal foundations to create opportunities for participation and integration, but also to recognise direct concerns of migrants and “ensure their rights to age in the way they want” ([6], 41). In Germany, older immigrants are characterised by opportunities and challenges to active ageing [7, 32]. On the one hand, families, ethnic communities and networks are presented as an important resource to self-support in older age. On the other hand, lack of health access, health literacy, and political participation act as barriers to an active and healthy environment for older immigrants [7]. Furthermore, Strumpen [33] concludes in her research on older Turkish immigrants in Germany that the idea of active ageing needs to be understood as a cultural phenomenon, which does not accurately represent older immigrants' expectations and thus presents a gap between policy, research, and older migrants' preferences.

Despite cultural and socioeconomic differences between the majority society and the population with a migration background, it is important not to overemphasise differences and to construct older migrants as “Others” in society. Thus, Torres [34] reminds us that socioeconomic challenges should not exemplify migrants as a social problem group. Moreover, the recognition of “foreign” cultural differences and cultural preferences should include an acknowledgement of own cultural biases and expectations. Therefore, in addition to describing cultural aspects of older immigrants, there must be a recognition of the German majority culture. Ageing is culturally specific and active ageing frameworks cannot be considered without cultural influences, which can exclude or neglect the needs of minority populations.

### 2.2. Individual Responsibility in Older Age

Active ageing is just one discourse strand in the wider ageing discourse. While this concept does not summarise the entirety of ageing discourses, it provides an insight into the discursive constructions of ageing, older age, and responsibility over time. Active ageing as a policy tool highlights the ideal perspective of how to support a healthy and active ageing population, looking both at the responsibility of the individual and society [5]. The discourse encompasses a shift from a problem perspective to the potentials in older age, by highlighting older people as an important resource for society and by emphasising ageing (actively) as an individual responsibility. The emphasis on activity stems from a long tradition of associating passivity and dependency as a weakness and ageing as a time of deficiency unless activity and independence can be sustained for as long as possible. Thus, the responsibility to keep active is the responsibility to keep independent [35]. Rüegger [35] questions the association of passivity and dependence as a weakness; instead, he argues that both autonomy and activity, as well as passivity and dependence, are normal human phenomena and that life continuously takes place between these poles. In the neoliberal context of active ageing, the strive for autonomy and independence is predominantly framed as an individual one, with collective and comprehensive public commitments taking a secondary role [3, 4].

### 2.3. Discourses on Health Services/Access

Examining discourses on ageing in the health care context offers an opportunity to analyse knowledge claims on meanings of ageing and health, perceived problems and their causes, responsibilities in society, and power effects, which translate into real health, care, and welfare practices. Discourse, as theorised by Foucault, describes a social system and social practice which creates knowledge and meaning, producing social facts [36]. These knowledge constructions become embedded in as well as performed by institutions, media, and social bodies. In the case of active ageing, the associated meaning of ageing well has been proclaimed by political institutions, media discussions, and research to produce a knowledge system where activity and independence in older age are positively connected with health and societal potential [37]. Furthermore, active ageing emphasises the self-governance of the individual, who is responsible to keep active, healthy, and independent [10, 37]. Discourses, therefore, affect individual and societal responsibility and the services available. Besides, the individual responsibility to stay healthy requires access to health-promoting resources, which is not equally available and has been shown to be lacking for migrant citizens [28].

## 3. Methodology

The study follows a sociology of knowledge approach to discourse analysis. This research framework combines theoretical aspects of the sociology of knowledge tradition and the microperspective of symbolic interactionism with Foucauldian perspectives on power and knowledge [38]. These theoretical foundations allow the researcher to analyse the actors' role in discourses and the (re)performance of discourses while being aware of the structures and power relationships in a discursive field. Using these interpretative sociological lenses provides a toolbox for analysing discursive constructions of knowledge on older immigrants. This study particularly aims to investigate how problems, their solutions, and associated consequences are raised in the literature and have changed over time. The discourse on older immigrants takes place on different societal levels spanning from everyday conversations to media, political, and academic arenas [39]. In this field of ageing, migration, and care, institutional actors such as political, healthcare, or ageing- and migration-related organisations engage in a specialist discourse, which is closely associated with academic discussions. Accordingly, discursive events are situated in the meso-/macroarena of political and organisational actors, which overlap with the scientific articulations and actors in this specialised field.

### 3.1. Data Collection and Sampling

Since 1995, the governmental report on foreigners in Germany addresses specifically the situation of older foreigners. Since 2000, the relationship between migration and ageing became a recurrent topic in government reports on families, the ageing population and migration/integration [40–42]. This surge of interest is also reflected in the scientific literature [43, 44]. However, some researchers have explored the topic of older age among the migrant population in Germany before the year 2000 [45]. Due to the rising interest in this topic in the last two decades, the literature search spans from the year 2000 onwards up to December 2019. The data collection followed the SKAD approach to include maximum and minimum contrasting documents. First, political reports were collected going through government reports published on the topics of ageing, migration, family, and voluntary work. Second, the search strategy included political reports, guidelines, and reports from political, public, religious, healthcare, or social organisations active in the fields of ageing and migrations. Thirdly, the data corpus was completed by other grey literature from healthcare or migration-related magazines or project reports. The online searches were performed using search terms that address older/ageing (im)migrants, foreigners, or people with a migration background together with keywords such as care, health, intercultural opening, elderly support (*Altenhilfe*), or health insurance. It has to be noted that the term “older (im)migrants” represents a heterogeneous population from numerous countries of origin, having migrated at different points of time at different ages and for a variety of reasons. Therefore, the search strategy on “older migrants”, “elders with a migration background”, and “ageing and migration” includes different definitions of who constitutes “older immigrants in Germany”.

The search strategy led to 43 documents for analysis. The data corpus consists of government reports (12); reports and articles by political foundations or charity and welfare organisations on the topic of social support, health care, and long-term care (12); federal state, municipal, and city government publications (10); texts by migration and health-related magazines (6); and documents by forums and working groups (3). All texts included in the analysis are publicly available online and written for an informed but not just academic audience.

### 3.2. Analysis

The project examines the discursive constructions at the intersection of ageing/older age and migration, including associated challenges and potentials as well as public or institutional services. Subject and speaker positions are identified to highlight different roles and practices within the discourse. The concept of phenomenal structures helps to examine themes by investigating associated elements such as problem constructions, responsibilities, course of actions, consequence, value implications, and the positioning of social actors [38]. The method of analysis draws upon grounded theory strategies; this includes creating codes to discover themes, constant comparison of codes and interpretations, and memo writing [46]. Furthermore, the grounded theory approach was used together with SKAD tools such as examining at a minimum and maximum contrasting examples using both fine and larger analysis techniques to assess the data corpus. Coding was carried out using the MAXQDA software or done by hand for detailed analyses.

## 4. Results

Older immigrants, in the documents analysed in this study, are predominantly described to be above 60-65 and to have lived in Germany for several decades. Documents from 2000 to 2005 focus more specifically on older foreigners. This definition changes to include older people with a migration background and mostly a personal migration experience after 2005. Largely, it is recognised that older immigrants are a heterogeneous group from various countries of origin with different life circumstances and social responsibilities. However, the attention mainly centres on two main migration movements, first, the former guest workers and, second, late repatriates (ethnic German resettlers). With the emphasis on former guest workers, the culture of immigrants is generalised as Southern, family-oriented, and traditional. Thus, despite the acknowledgement of the diversity of immigrants, a homogenous description of migrant communities and preferences is presented. Therefore, the results pertain to older immigrants in general with some discourse strands referring specifically to former guest workers.

### 4.1. Subject Positions and Phenomenal Structure

Over the span of two decades, two major subject positions emerge that can be associated in their phenomenal structures.  Table 1 depicts values, perceived problems and their causation, and associated informal and public/municipal responsibilities related to these positions. Undeniably, utterances also fall between these positions and discourse events can refer to both positionings. Nevertheless, these subject positions present two key viewpoints on older immigrants. The traditional position is somewhat more common in earlier documents and the municipality position more frequent in more recent publications. The results presented in the following demonstrate the shift from more traditional to integrated perspectives on older immigrants, which is partially reflected in the time of publication.

### 4.2. Active in Private–Passive in Public

Older immigrants appear to be more passive in society with few remarks on leisure time activities, political participation, and voluntary work. Accordingly, ageing is depicted as an ethnic withdrawal from society. Also, integration is described as “particularly difficult at an advanced age” and the motivation of integration is assumed “to disappear in old age” ([47], 20). The passivity of older immigrants is further marked out by existing language deficits, which obstruct participation in society [48]. By contrast, activity is predominantly associated with commitments in the informal sphere, specifically, care work within the extended family. Furthermore, the emphasis is frequently on social groups, such as the family or ethnic community, but seldom on the individual. Therefore, the individual older immigrant appears passive while the community is praised for its self-support. For instance, the “German Report on Ageing” ([48], XIII) approves how “families—partly supported by outpatient care services—do a great job in caring for their relatives.” These forms of activity are seen as great potentials, which need to be recognised more to move away from negative stereotypes ([49], 27). For example, circular migration between sending and receiving country represents a migrant-specific resource that allows an active lifestyle in old age, keeping in touch with relationships in both countries and leading to higher life satisfaction [50]. These associations of being active within the family and the close social circle and passive in the public sphere is further emphasised for older immigrant women. Women are frequently associated with the household, the ethnic community, and their social responsibilities within these social structures.

*The social networks in the ethnic colony can have many functions, e.g. in the field of lay medical systems and the reciprocal support of women, which is important for the care of the elderly in families. ([* 41 *], 499) (All quotes were translated by the author and were discussed with another German and English-speaking researcher to ensure a close translation of the original texts.)*

As the quote shows, the emphasis is on the role of the lay medical knowledge and the cooperation between women in the informal sphere. On one hand, activity is emphasised and promoted as a valuable resource for independence. On the other hand, activity is presented as restricted and segregated from society, distinct to the private realm of older immigrants, which generates an image of passivity. In line with general discourses on active ageing, being active presents the prerequisite of independent ageing, which is constructed as the overarching aim. Therefore, the goal is to support activity and social responsibility to ensure autonomous, independent, and self-supported ageing as a solution.

### 4.3. Collective “Ethnic” Responsibility

In the family, the close social circle, and “ethnic” networks of older immigrants, activity and support are constructed as a collective “ethnic” responsibility. Above all, the (extended) family or family associations (*Familienverbände)* ([40], 121) play an essential role in the discourses on older immigrants. Cultural values are seldom related to a specific cultural background or region of origin but generally describe the perceived cultural difference of immigrants to German society. These cultural aspects, associated with a migration background in older age, highlight the value of familial cohesion, intergenerational support, and the significance of the extended family. This includes the expectations on relatives that care preferably takes place within the family. Solidarity and intergenerational support among the migrant community is introduced as a cultural and traditional value. Moreover, it is explained that immigrants do not want “to be a burden” ([51], 4) of society and have high expectations of support structures within their communities [52]. Older immigrants live predominantly in larger family households due to cultural and financial reasons, which facilitates familial support. The importance of family support is a dominant discourse theme appearing in most documents. By stating the cultural significance of familial support, the expectation and responsibility for migrant relatives to care for each other becomes accentuated by discourse actors, i.e., political institutions, health and welfare organisations, and migrant organisations. This emphasis on self-supporting values stresses the obligation of older immigrant to care for each other, as it is shown in the following quote from conference proceedings of a symposium on migration and ageing:

*These people grew up in a different culture, where elderly people can expect help but where no demands are made. They grew up in a culture of informal help and family solidarity. Official services and institutions are foreign to them. That means that this is a radical change for them. ([* 52 *], 47)*

Though, it is recognised that despite the efforts within families, additional support may be required as there may be a risk of overburdening the family by providing long-term care for relatives [53, 54]. Therefore, reports conclude that there needs to be additional care assistance, for example, in form of outpatient care, to sustain the collective support within private networks.

Beyond the family, an additional emphasis lies in the informal structures of older immigrants. The potentials and resources of immigrants are found in networks, ethnic communities, and neighbourhoods, which are characterised by high informal engagement, self-support, and help networks [49]. Yet, these forms of social activity are depicted as informal and private and not as formal voluntary work. Voluntary work or social engagement (*Ehrenamt*) is described as a foreign concept for older immigrants:

*In many countries of origin, societal structures are also much less developed than in Germany, and there are sometimes few equivalents in other languages for the German term ‘Ehrenamt'. The idea of getting involved in formal (association) structures for people with whom there are no family or neighbourly ties is, therefore, a strange idea, especially for the first generation of immigrants. ([* 55 *], para. 8).*

Thereby, the difference between informal activity and voluntary work in associations is frequently noted, but it remains undefined what counts as informal support and what as societal participation. Moreover, migrant organisations are portrayed as another form of an informal social resource. These are presented as outside of traditional German voluntary work. Though migrant organisations are embedded in official structures, they appear as being informal and separate from German voluntary organisations. The emphasis on family cohesion and the informal (“ethnic”) structures of older immigrants leads to a presentation of migrant communities being distant and separate from the majority society. The continuous focus on informal, ethnic, cultural, or family-related therefore constructs a line between the collective support of older immigrants and other services that exist outside in the public sphere.

### 4.4. Opening Private Support Systems and Connecting Existing Structures

As shown by the emphasis on the collective support structures of migrants, it appears that there is a boundary between informal structures and public engagement. However, the discourse also depicts a shift towards opening informal networks to make social engagement public and, thus, more widely accessible, especially in later documents. This change is also demonstrated in the description of informal structures. The nature of these networks is constructed differently over time from descriptions of segregated “neighbourhoods” or “ethnic colonies” to more openly described “communities” and “structures.” This also includes a shift from acknowledging familial and neighbourhood networks to a call for supporting the cooperation of local communities with public services. Overall, ethnic networks represent private and inaccessible resources, whereas institutional or publicly organised support is presented as cultureless and open for all members of society. Consequently, the promotion of migrant organisations and networks is increasingly encouraged with the aim to connect these to the public sphere.

The aim is to open existing informal structures, connect them with public services, and bring them from private, inaccessible spheres to the public. As a result, existing support structures need to be recognised and constructed as open and accessible. While self-supporting resources exist, they are not sufficient. Thus, opening and bridging the informal with migrant organisations and public services becomes a strong effort.

*At the same time, the extensive informal support networks of older immigrants need to receive more attention. The existing assistance services and support systems should be opened for these forms of self-organization. ([* 48 *], 91)*

Besides, the connection of structures includes linking existing welfare services such as actors in senior citizen work, geriatric care, integration work, and migrant organisations. The aim is to connect the topics of older age and migration and to improve cooperation among actors, institutions, and welfare amenities as thus far services have seldom overlapped. Even for migrant organisations, ageing is an underaddressed issue that is still gaining importance. This opening of the informal includes bridging the communities of older immigrants with existing municipal services. To achieve this, migrant organisations play an important role to bridge migrant communities with general society with the use of migrant representatives. Through emphasising the image of the bridge from the immigrant community to the German majority society, migrant communities, again, appear as detached from the general public. In addition, immigrants are characterised by being difficult to contact and to welcome in existing services. Therefore, persons with a migration background themselves need to act as a link between migrant and nonmigrant [47, 49, 56, 57]. Here, it is the migrant background that is the unifying feature, not the cultural similarities. Thus, it seems as understanding the socioeconomic situation may be more relevant than overcoming cultural differences. On the one hand, the connection of services aims to further support immigrants; on the other hand, it also emphasises the expectation that older people contribute to society, not just in private but also in the public sphere.

### 4.5. Shifting Responsibility to the Municipality

In more recent documents, the importance of the municipality appears as a space for interaction and bridging various actors in the field of migration, ageing, and health/care. Migrant structures should be opened to be accessible for the municipality, and the connection of existing services is suggested on a municipal level. The municipality becomes the community, which refers to all citizens as an inclusive entity independent of migrant background. Social issues are, therefore, municipal issues and not migrant-specific problems. This includes the promotion of more culturally sensitive and intercultural approaches in prevention, health promotion, and long-term care as an ongoing process within municipal services. However, with the focus on the municipal community, the responsibility of addressing these challenges lies in the entire community. The focus on the municipality, therefore, comes with a shift from the social responsibility of immigrants to the responsibility of the local community. This responsibility produces the image of the municipality as a social actor taking responsibility and solving its problems:

*In order to improve the living situation of senior citizens with a migrant background, it is important that municipalities take demographic change into account when developing their intercultural concept and that their services check their relevance for migrants of senior age and expand them if necessary. ([* 58 *], 13)*

Thereby, it remains elusive what and who the municipality represents. The image of the municipality refers to the local community and the social responsibility of people living in this community. Thus, the responsibility remains close to older immigrants. This emphasis on the local also demonstrates that the challenges of older immigrants are due to the services and support available in the local community and are more distantly related to issues on a national level. The shift to the municipality represents an inclusive local society and the coming together of various welfare services. However, a public or national responsibility on providing services for the older generation remains vague.

## 5. Discussion

This discourse analysis investigates ageing and specifically the active ageing discourses in the political and institutional arenas at the intersection of ageing and migration. The discourse on older immigrants includes elements of the active ageing discourse, including a call for individual responsibility and volunteer work. Yet, in the context of older immigrants, the focus lies not on the individual but social groups, i.e., ethnic communities, families, and migrant networks. These social groups are expected to support each other but also to make existing social structures accessible and to connect with public services. Moreover, the discourse has shifted increasingly to the responsibilities of, and reciprocal support within, the municipality. Consequently, two opposing subject positions of older immigrants emerge: first, the traditional, family-oriented immigrant and, second, immigrants as municipal citizens and “bridging agents” connecting existing services.

The paper analyses the German discourse on ageing and migration, which is influenced by the German healthcare and welfare service landscape. The study demonstrates how stereotypical representations of immigrants become included in the active ageing framework and therefore shape the opportunities for ageing immigrants. Thus, the presented connection of migrant discourses and their effect on ageing initiatives is an example of active ageing discourses in Europe and highlights the challenges of creating inclusive active ageing policies. Furthermore, the results display how knowledge around immigrants and knowledge on ageing influence each other and how these systems of knowledge become reconstructed within the active ageing frameworks.

### 5.1. Between Activity and Passivity

In the discourse on older immigrants, activity is communicated differently than in the general discourse on (active) ageing. Activity and passivity are understood in the context of culture, ethnicity, and public accessibility. Activity in the informal and “ethnic” community does not, thereby, gain the same praise as being formally involved in social engagement. Instead, self-support in the migrant community appears inaccessible. There is, however, one exception: care activity in the family is continuously praised as a cultural value and responsibility. What is considered an activity is an ongoing question regarding active ageing. In the data, what counts as active or passive seems to be measured on its value for society. As Foster and Walker [3] remark in this issue, narrow definitions of activity pose a threat as they reduce the variety of actions to productivity (such as paid work). Furthermore, Minkler and Holstein [59] underline that pleas for civic engagements should not undermine private acts of sustenance and nurture by highlighting the potential of volunteering and social engagement for society's social structures. Hence, activity should not be defined by its value for the majority society but should encompass “all meaningful pursuits which contribute to individual well-being” ([3], 6), which is particularly important with increasing diversity in older age. As Strumpen ([33], 315-316.) remarks in her research on older Turkish immigrants in Germany, active ageing should be understood as a cultural phenomenon and as a manifestation of the ageing culture in Germany. Therefore, active ageing is not without cultural preconceptions which influence how activity and ageing well are conceptualised and promoted [34, 60]. Recognising active ageing's cultural biases provides a necessary foundation to develop cultural sensitivity within active ageing and other ageing concepts.

### 5.2. Active Ageing as a Form of Integration

Ideally, active ageing should function as an inclusive framework to enhance age-related integration throughout the life course, leading to an inclusive environment to facilitate participation in the community in older age [61]. At the same time, older people are expected to be engaged and active for as long as possible and therefore remain integrated in public life [4]. For former guest workers in Germany, integration measures were limited for decades because their stay was expected to be temporary [62]. This lack of integration raised barriers for immigrants to access services, and these barriers persist due to language difficulties, discrimination experiences, and information deficits [62–64]. Accordingly, opening existing public services, linking organisations, and migrant-specific initiatives could serve as an important step towards establishing an inclusive environment. Creating inclusive structures in older age holds the potential to create both age and culturally sensitive services for older immigrants. With regard to former guest workers, older age is constructed as a second chance for integration missed earlier in life. However, integration should not be framed as an individual responsibility; instead, it needs to be addressed on a macro level going beyond the local, municipal districts [65].

### 5.3. From Ageing as ‘the Other' to Municipal Responsibility

The SKAD approach, by examining knowledge constructions and subject positions, enables exploring the positioning of older immigrants, which demonstrates the role of Othering language in the discourse. In the first subject position, which centres on older immigrants as traditional, family-oriented, and situated in ethnic networks, older immigrants are constructed as a distinct social group who appear as the Other in contrast to older nonmigrants. Similarly, Torres [34] observes that older migrants in Sweden are othered but considered a social problem group in the discourse on elderly care and policy. In the German context, Hahn [65] discusses how the ethnicity and culture of older immigrants have been a defining feature since the start of the discourse on ageing and migration in Germany. This perception of difference and Otherness, highlighting “us” versus “them,” continues in social work and manifests on the city district level [65]. Thus, the focus on ethnicity and cultural difference conceptualises ageing with a migration background as inherently different. That is precisely why the shift to responsibility within the municipality is a curious trend as it positions older immigrants as part of the municipality. This diverts the focus from “ethnic networks” to the local community. The discourse, however, remains elusive on the definition of the municipality and who it entails. Ultimately, the shift to opening and connecting existing services in the municipality underscores a recognition of environmental and public structures, yet it also places the responsibility to contribute and stay active with the citizens.

### 5.4. Health in the Active Ageing Paradigm

The discourse on older immigrants in Germany demonstrates the ongoing dynamic on conceptualisations of ageing and ageing well. The relationship between activity and health is one aspect of the active ageing discourse that is under discussion; depending on the definition of ageing well, good health can be a means and a goal of active ageing [66]. A Health Science perspective can be beneficial, first, to recognise the effect of the life course on health and activity later in life [3] and, second, to consider the role of social determinants of health and environmental factors [66]. This perspective is particularly important in the context of migration, where integration measures have been limited and accessibility to existing services is lacking [11, 62]. While health risks in the past have been extensively explained with socioeconomic determinants in the data, strategies to develop health promotion in older age remain a side topic. How active ageing policies can promote health needs to be discussed on an institutional, political level to not rashly rely on an undertheorised connection between activity with health and well-being [67]. Instead, official action and welfare services must be clear and not appear as agents eluding responsibility, as the municipality is presented in the discourse. Supporting structures must be developed to promote an active and healthy lifestyle throughout the life course and specifically for the older population.

### 5.5. Limitations

The data collection only included online available publications, which limits the scope of the discursive events. With the increase in digitalisation, the search strategy might have led to more recent publications and fewer documents from the early 2000s, which could limit the variety of texts included. However, both government documents and organisation reports are published throughout the entire period and were included in the data corpus. Another limitation is that various speakers use different terminology to refer to older immigrants, which refer to different age groups and, in some cases, focus specifically on older foreigners, former guest workers, or Southern European immigrants. While this paper sought to address this lack of clarity, generalisations in the discourse and the associated knowledge constructions of age, migration background, and terminology, the term “older immigrants” in the documents analysed here and therefore in the results remains inadequately defined.

## 6. Conclusions

This paper demonstrates how active ageing is conceptualised in the context of older immigrants in Germany. The emphasis on informal and collective support structures leads to understanding active ageing as a collective responsibility for older immigrants, with families expected to provide long-term care for their members. Furthermore, there is a discourse shift to solutions in the municipalities, where existing health care, long-term care, migration, and age-related services can connect and provide a holistic support structure for older immigrants. Older immigrants would therefore become engaged active citizens in their municipality. Thus, the active ageing discourse at the ageing and migration nexus focuses on collectivity instead of individual responsibility and emphasises the cultural and ethnic differences regarding ageing preferences. Rather than highlighting the difference between older immigrants and constructing immigrants as the “Other” older population, cultural biases need to be acknowledged in the active ageing framework. Demographic change will include an increase in the diversity in the experience of ageing, due to the manifold reasons for migration, the multiplicity of countries of origin, gender, sexual orientation, and dis/ability. The inherent ageism in ageing discourses has been extensively criticised in Social Gerontology, yet additional forms of discrimination and Othering need to be addressed by developing diversity-sensitive and inclusive ageing frameworks. In so doing, active ageing can play a significant role in realising the social inclusion of older immigrants.

## Figures and Tables

**Table 1:** Phenomenal structure and subject potions of older immigrants.

Interpretative pattern	Social responsibilities of older immigrants are a resource for self-supporting ethnic networks	Social responsibilities of older immigrants are a resource for society
Subject position	(i) Older immigrants are traditional and family-oriented; therefore, activity and social engagement take place in extended families and within extensive ethnic networks. (ii) Because of cultural differences, older immigrants age differently.	(i) Older immigrants are a heterogeneous group (culturally, ethnically, economically), often having experienced socioeconomically challenging and discriminatory living conditions. (ii) Their social networks include the (extended) family, migrant organisation, and the municipality.

Values	(i) Family values are based on intergenerational cohesion, i.e., care in the family is best; recognition for delivering care within the families. (i) Traditional or cultural values provide as a sense of belonging (“identity anchor” ([47], 5))	(i) Promoting responsibility for and participation in the community. (ii) Achieving independent living in old age. (iii) Overcoming a deficit perspective on older migrants and strengthening potentials.

Perceived problems and causation	(i) Increased health risks compared to the nonmigrant older population due to low-paid and high-risk employment, short time in employment, and financial insecurity. (ii) Changing family structures challenge the provision of care by family members.	
	(i) Migration leads to disadvantaging living situations (migration as a risk factor, e.g., due to psychosocial stress). (ii) Lack of knowledge on services and authorities in the health and social sector as well as language barriers. (iii) Previous expectation that older immigrants will migrate back to the country of origin in old age. (iv) Ethnic networks are not accessible, segregated from society.	(i) Older immigrants live predominantly in discriminatory and low-resource socioeconomic circumstances. (ii) Municipalities and their health and social services are not adapted to cater for older immigrants; care, health promotion and social services are underutilised. (iii) Experiences with discrimination in public service institutions. (iv) Lack of accessibility of existing social/health structures due to language barriers, limited integration, and information deficits.

Older immigrants' responsibilities	(i) Family is a central resource for support and care. (ii) Important services are carried out in the (ethnic) communities. (iii) Older immigrants need to familiarise themselves with state services.	(i) Social involvement in the municipalities is expected. (ii) Migrant organisations need to be opened and accessible to society. (iii) Immigrants can fulfill bridging positions between ethnic/private networks, migrant organisations, and municipal services.

Public/municipal responsibilities	(i) Support of family and extrafamily networks and expansion of support services. (ii) Assistance for long-term care in families with outpatient care. (iii) Developing intercultural or culturally sensitive approaches in health, care, and welfare services.	(i) Improving access to public/municipal services (i.e., health promotion, ambulatory, and stationary long-term care). (ii) Developing intercultural approaches in institutions such as culturally sensitive care; avoiding parallel structures of migrant/nonmigrant clientele. (iii) Better networking of existing actors, resources, and services; connecting private and public services; recognising informal work of older immigrants; and providing support for independent living.

## Data Availability

The discourse analysis data used to support the findings of this study are available from the corresponding author upon request.

## Supplementary Materials

Supplementary Materials The supplementary data provides the list of documents included in the data corpus by publication type.
