# Supplementary material for: Activity and Social Responsibility in the Discourse on Health Care, Long-Term Care and Welfare Services for Older Immigrants
Source: Biomed Res Int. 2021 Jul 30;2021:5241396. doi: 10.1155/2021/5241396 (PMC8346296; doi:10.1155/2021/5241396)
Supplement: Supplementary Materials — The supplementary data provides the list of documents included in the data corpus by publication type. [file 5241396.f1.docx]

# Supplementary Material

## List of sources included in the data corpus

### Government reports and guidelines

[1] BMFSFJ, “Selbstständige Lebensführung älterer Migrantinnen und Migranten in Deutschland - Handlungsbedarfe und strukturelle Ansatzpunkte,” 2016.

[2] “Siebter Altenbericht zur Lage der älteren Generation in der Bundesrepublik Deutschland: Sorge und Mitverantwortung in der Kommune,”. BMFSFJ. Deutscher Bundestag, 2016, Drucksache 18/10210.

[3] “Gesundheit älterer Migrantinnen und Migranten im Blickpunkt,” https://www.programm-altersbilder.de/meldungen/aeltere-meldungen/meldungen-2015/100615-gesundheit-aelterer-migrantinnen-und-migranten-im-blickpunkt.html.

[4] Die Bundesregierung, *Migranten im Alter liebevoll betreut*: *Pflege für Menschen aus anderen Kulturen*, 26.08.2014.

[5] “10. Bericht,”. Beauftragte der Bundesregierung für Migration, Flüchtlinge und Integration, 2014, Drucksache 18/3015.

[6] “9. Bericht,”. Beauftragte der Bundesregierung für Migration, Flüchtlinge und Integration, 2012.

[7] “Sechster Bericht zur Lage der älteren Generation in der Bundesrepublik Deutschland: Altersbilder in der Gesellschaft,”. BMFSFJ. Deutscher Bundestag, 2010, Drucksache 17/3815. Berlin, 2018.

[8] “8. Bericht,”. Beauftragte der Bundesregierung für Migration, Flüchtlinge und Integration, 2010, Drucksache 17/2400.

[9] “6. Bericht,”. Beauftragte der Bundesregierung für Migration, Flüchtlinge und Integration, 2005.

[10] “Fünfter Bericht zur Lage der älteren Generation in der Bundesrepublik Deutschland,”. BMFSFJ, 2005, 16/2190.

[11] “4. Bericht,”. Beauftragten der Bundesregierung für Ausländerfragen, 2002.

[12] “Sechster Familienbericht: Familien ausländischer Herkunft in Deutschland Leistungen - Belastungen - Herausforderungen und Stellungnahme der Bundesregierung,”. Deutscher Bundestag, 2000.

### Political foundations, charity and welfare organisations

[1] E. Olbermann, “Das Alter wird bunter: Lebenslagen älterer Menschen mit Migrationshintergrund und Handlungsbedarfe für Politik und Gesellschaft,”. Friedrich Ebert Stiftung, 2013, vol. 41. Routledge, Bonn, http://library.fes.de/pdf-files/wiso/10188.pdf, Accessed February 27, 2018.

[2] H. Baykara-Krumme, “Ältere MigrantInnen in Deutschland – ein Überblick zur demographischen Entwicklung,” in *Altern in der Migrationsgesellschaft,* Heinrich-Böll-Stiftung, Ed., pp. 9–11, 2012.

[3] H. Baykara-Krumme, “EinwanderInnen und ihre Familien: Generationenbeziehungen in der Lebensphase Alter,” in *Altern in der Migrationsgesellschaft,* Heinrich-Böll-Stiftung, Ed., pp. 22–26, 2012.

[4] S. Huth, “Bürgerschaftliches Engagement von älteren MigrantInnen,” in *Altern in der Migrationsgesellschaft,* Heinrich-Böll-Stiftung, Ed., pp. 27–32, 2012.

[5] E. Olbermann, “Gesundheitliche Situation und soziale Netzwerke älterer MigrantInnen,” in *Altern in der Migrationsgesellschaft,* Heinrich-Böll-Stiftung, Ed., pp. 33–37, 2012.

[6] I. Tucci, “Die Einkommens- und Wohnsituation älterer MigrantInnen,” in *Altern in der Migrationsgesellschaft,* Heinrich-Böll-Stiftung, Ed., pp. 12–17, 2012.

[7] Y. Yılmaz-Aslan, P. Brzoska, and O. Razum, “Gesundheitsförderung und Prävention bei älteren Menschen mit Migrationshintergrund,” in *Altern in der Migrationsgesellschaft,* Heinrich-Böll-Stiftung, Ed., 38-41, 2012.

[8] G. A. Miethke, “Jetzt reden wir! Perspektiven und Interessen älterer Zuwanderinnen und Zuwanderer im bürgerschaftlichen Engagement,”. Friedrich-Ebert-Stiftung, Forum Politik und Gesellschaft, 2011, http://library.fes.de/pdf-files/do/08210.pdf, Accessed January 4, 2019.

[9] M. May, “Ältere Migrant(inn)en im Quartier – Stützung und Initiierung von Netzwerken der Selbstorganisation und Selbsthilfe,”. Stiftung Mitarbeit, 2010, no. 4, https://www.buergergesellschaft.de/fileadmin/pdf/gastbeitrag_may_100305_end.pdf, Accessed January 4, 2019.

[10] F. Palecek, “Ältere MigrantInnen - Soziale Lage und Gesundheit,” in *Migration & Gesundheit,* Heinrich-Böll-Stiftung, Ed., pp. 36–43, 2009.

[11] “Leitfaden für eine interkulturelle Seniorenarbeit: Das Konzept der AWO Berlin,”. Arbeiterwohlfahrt Landesverband Berlin e.V., 2004. Berlin, http://www.begegnungszentrum.org/uploads/media/AWO-Leitfaden-Interkulturelle-Seniorenarbeit.pdf, Accessed February 5, 2019.

[12] D. Baric-Büdel, *Impulsreferat: Interkulturelle Öffnung der Altenhilfe der AWO*, 2002.

### Federal state, municipal and city government publications

[1] L. de Paz Martínez, T. Koepf, S. Reckhaus et al., “Soziale Hilfen,” in *Möglichkeiten der Gestaltung migrationsbedingter Vielfalt*: *Praxishandreichung zur interkulturellen Öffnung in Kommunalverwaltungen,* Institut für Sozialpädagogische Forschung Mainz, Ed., pp. 43–50, Mainz, 2019.

[2] Landesintegrationsrat Nordrhein-Westfalen, “Älteren Menschen mit Migrationsgeschichte den Zugang zu Pflege- und Altenhilfe erleichtern und ihre Lebensleistung würdigen,” https://landesintegrationsrat.nrw/stellungnahme-des-landesintegrationsrates-nrw-zum-antrag-der-spd-landtagsfraktion-aeltere-menschen-mit-migrationsgeschichte-den-zugang-zu-pflege-und-altenhilfe-erleichtern-und-ihre-lebensleistung-wu/.

[3] “Gesundes Altern in vielen Welten: Gesundheit von älteren Zugewanderten,”. Gesundheit Berlin-Brandenburg e. V. Koordinierungsstelle Gesundheitliche Chancengleichheit Brandenburg, 2016. Potsdam, https://www.slaek.de/media/dokumente/01/03Empfehlungen/2016-11_Gesundes_Altern_in_vielen_Welten-web.pdf, Accessed January 27, 2019.

[4] A. Kronenthaler, H. Hiltner, D. Müller et al., “Eine Handreichung zur medizinischen und pflegerischen Versorgung von älteren Migrant_innen: Ältere türkische/ türkischstämmige Migrant_innen der ersten Gastarbeitergeneration im Gesundheitswesen,”. Care for Elderly Migrants (CarEMi), 2016, http://www.caremi.de/Handreichung-CarEMi-de.pdf, Accessed May 27, 2019.

[5] “Älter werden in München: Kurzbericht,”. Landeshauptstadt München, 2015. München, Accessed November 3, 2018.

[6] “Immer mehr Menschen mit Migrationshintergrund in der Plege,”. Baden-Württemberg.de, 11/27/2014, https://www.baden-wuerttemberg.de/de/service/presse/pressemitteilung/pid/immer-mehr-menschen-mit-migrationshintergrund-in-der-pflege/, Accessed March 6, 2020.

[7] E.-M. Pietzcker, “Alter, Migration und Gesundheit: Leitfaden und Projektdokumentation aus dem Mehrgenerationenhaus Nürnberg-Schweinau,”. Mehrgenerationenhaus Nürnberg-Schweinau, 2012, https://www.nuernberg.de/imperia/md/seniorenamt/dokumente/programmhefte/leitfaden_alter_migration_gesundheit.pdf, Accessed February 5, 2019.

[8] K. Lis, E. Olbermann, C. von Koenen et al., “Gesundheitsförderung für ältere Menschen in sozial benachteiligten Lebenslagen im Setting Stadtbezirk: Ein Leitfaden mit praktischen Handlungsempfehlungen,”. Forschungsgesellschaft für Gerontologie e.V. and Institut für Gerontologie an TU Dortmund, 2011. Dortmund, https://www.in-form.de/fileadmin/user_upload/Praxishandbuch_Aelter_werden_in_Eving.pdf, Accessed August 23, 2019.

[9] “Handlungskonzept zur Integration älterer Migrantinnen und Migranten in das Netz der Altenhilfe in Hamm,”. Oberbürgermeister der Stadt Hamm. Fachbereich Jugend, Gesundheit und Soziales, 2010. Hamm, https://www.hamm.de/fileadmin/user_upload/Medienarchiv/Gesundheit_Soziales/Migration/Dokumente/52-522-523/52-523_Handlungskonzept_Integration.pdf, Accessed February 5, 2019.

[10] H. M. Martinez, “Anforderungen, Chancen und Möglichkeiten einer interkulturellen Öffnung der Altenarbeit – Die Rolle der Migrantenselbsthilfeorganisationen,” in *Auch Migranten werden alt!* *Lebenslagen und Perspektiven in Europa,* Forschungsgesellschaft für Gerontologie e.V., Ed., pp. 73–77, Unna, 2004.

### Magazine and online articles

[1] Redaktion Rechtsdepesche, “Welche Pflege sich türkischstämmige Senioren wünschen,” https://www.rechtsdepesche.de/welche-pflege-sich-tuerkischstaemmige-senioren-wuenschen/.

[2] J. Schmidt and Kuratorium Deutsche Altenhilfe, “Pflegeangebote für ältere Migranten: Versorgung kultursensibel ausrichten,” 3/1/2018, https://www.apotheken.de/news/article/pflegeangebote-fuer-aeltere-migranten/.

[3] U. Gasser, “Lebenssituationen von Senioren mit Migrationshintergrund,” http://www.migazin.de/amp/2013/04/23/lebenssituationen-von-senioren-mit-migrationshintergrund/.

[4] P. Brzoska and O. Razum, “Gleich gesund und gut versorgt? Zur gesundheitlichen Lage und Versorgung von Menschen mit Migrationshintergrund,” in *MDK Forum. Das Magazin der Medizinischen Dienste der Krankenversicherung*: *Migration und Gesundheit,* Medizinischen Dienst des Spitzenverbandes Bund der Krankenkassen e.V., Ed., pp. 5–6, 2012.

[5] F. Geisler, “Pflege von Menschen mit Migrationshintergrund. Geblieben und alt geworden,” in *MDK Forum. Das Magazin der Medizinischen Dienste der Krankenversicherung*: *Migration und Gesundheit,* Medizinischen Dienst des Spitzenverbandes Bund der Krankenkassen e.V., Ed., pp. 9–10, 2012.

[6] MDK forum, “Interview mit Ministerin Aygül Özkan. Integration statt Parallelwelt,” in *MDK Forum. Das Magazin der Medizinischen Dienste der Krankenversicherung*: *Migration und Gesundheit,* Medizinischen Dienst des Spitzenverbandes Bund der Krankenkassen e.V., Ed., pp. 7–8, 2012.

### Forums and working groups

[1] A. Zerche, “Älteren Migranten droht besonders Armut,”. Deutsches Institut für Altersvorsorge, 2018, https://www.dia-vorsorge.de/demographie/aelteren-migranten-droht-besonders-armut/, Accessed March 6, 2020.

[2] “Gesundheit und Pflege in der Einwanderungsgesellschaft: Stellungnahme und Handlungsempfehlungen der Migrantenselbstorganisationen zur gesundheitlichen Versorgung von Menschen mit Zuwanderungsgeschichte und Flüchtlingen.,” 2015, https://www.integrationsbeauftragte.de/resource/blob/72490/481452/d4e68c73dcbfead2442a802a6a2450eb/gesundheit-mo-papier-data.pdf.

[3] “Memorandum für eine kultursensible Altenhilfe: Ein Beitrag zur Interkulturellen Öffnung am Beispiel der Altenpflege,”. Forum für eine kultursensible Altenhilfe, 2009.
